# Biofilm overproduction enhances gastrointestinal stress tolerance and intestinal fitness in *Bacillus subtilis*

**DOI:** 10.1080/19490976.2026.2684066

**Published:** 2026-06-21

**Authors:** Lohith Kunyeit, Reeta Rao

**Affiliations:** a Department of Biology and Biotechnology, Worcester Polytechnic Institute, Worcester, MA, USA

**Keywords:** *B. subtilis* biofilm, simulated digestive fluids, *Saccharomyces boulardii*, bio-encapsulation, *Caenorhabditis elegans*, flocculation, soy milk

## Abstract

Microorganisms with health-promoting potential often experience substantial losses in viability and function due to stresses encountered during manufacturing and gastrointestinal transit. In this study, we investigate whether biofilm can be leveraged to enhance microbial resilience and functional performance. Using *Bacillus subtilis* as a model biofilm-forming bacterium, we examined strains with defined biofilm phenotypes: a biofilm-deficient mutant (*tasA eps*), a biofilm-overproducing mutant (*sinR*), and an isogenic wild-type control. These strains were evaluated across multiple functional benchmarks, including survival in simulated gastric and bile juices, thermotolerance, and intestinal bacterial colonization in the *Caenorhabditis elegans* model. Commercially available strains *Lactobacillus rhamnosus* GG and *Saccharomyces boulardii* were included as reference comparators. The biofilm-overproducing *B. subtilis sinR* strain demonstrated markedly enhanced survival under simulated gastrointestinal conditions and showed increased colonization within the *C. elegans* intestine. In contrast, the biofilm-deficient *tasA eps* mutant exhibited severe sensitivity to gastric stress and reduced the intestinal bacterial load. Furthermore, we demonstrate that cell-free *B. subtilis* biofilm can function as an effective bioencapsulation matrix. When used to encapsulate multiple probiotic strains, the biofilm matrix significantly improved their survival under acidic gastric conditions by neutralizing the environmental pH, indicating its broad potential for probiotic formulations and targeted gastrointestinal delivery. Overall, biofilms are traditionally studied for their roles in infection and antimicrobial resistance; however, their protective and adaptive traits may be repurposed for beneficial use. As an example of this concept, our findings show that *B. subtilis* biofilms enhance multiple functional and technological traits and highlight biofilm-based strategies as a promising platform for improving beneficial microbial robustness and the delivery of live biotherapeutics.

## Introduction

Probiotics are beneficial microorganisms capable of withstanding the harsh conditions of the gastrointestinal tract while remaining viable and metabolically active. Popular probiotics such as *Lactobacillu*s, *Bifidobacterium* species, and *Saccharomyces boulardii* have been widely used in the management of gastrointestinal disorders, including antibiotic-associated diarrhea, irritable bowel syndrome, and inflammatory bowel disease.^1-3^ These beneficial microbes are commonly delivered through dietary sources or as commercial formulations, typically in the form of lyophilized or spray-dried encapsulates packaged as capsules or sachets.^4^ Despite advances in formulation and delivery, probiotic products often experience a significant reduction in viability during oral consumption. This loss of viability is primarily attributed to exposure to digestive enzymes, gastric acid, and bile salts within the gastrointestinal tract. Additionally, technological challenges associated with the processing, handling, storage, and interactions with food components such as sugars, salts, and aroma compounds further compromise probiotic efficacy.^5^ ^,^ ^6^ Hence, several approaches have been developed to overcome these limitations, including alginate gelation, multilayer coatings, nanoencapsulation, and engineered probiotics, to improve survival and enable targeted gut delivery.^7^

Biofilms are structured communities of microorganisms that provide collective survival under adverse environmental conditions.^8-10^ Research on biofilms has traditionally focused on their role in infection and antimicrobial resistance, as pathogenic biofilms pose a major challenge to effective antimicrobial therapy and are associated with adverse clinical outcomes. In contrast, we propose that biofilms formed by beneficial microorganisms can be leveraged to enhance both biological efficacy and technological performance of probiotics. Biofilms possess intrinsic resistance to extreme environmental stresses including, acidic and alkaline pH, elevated temperatures, and oxidative conditions, making them advantageous for maintaining viable cell counts during exposure to harsh gastrointestinal conditions (e.g., gastric acid and bile salts) as well as downstream processing operations such as thermal treatment and freeze-drying.

*Bacillus subtilis* is a soil bacterium known for its ability to form biofilms in diverse environments, making it a well-established system for studying bacterial biofilm formation.^11^ In this study, we have used *B. subtilis* as a model probiotic bacterium to demonstrate the significance of its biofilm formation in probiotic applications. *B. subtilis* is already well established as a spore-forming probiotic because its spores can withstand adverse gastrointestinal conditions. In addition, studies have revealed that vegetative forms of several *B. subtilis* strains can survive the harsh conditions of the gastrointestinal tract.^12^ However, the biofilm lifestyle of *B. subtilis* may offer advantages beyond those of conventional spore- or vegetative cell-based probiotics through enhanced surface adhesion and colonization potential, modulation of host immune responses, and beneficial interactions with surrounding microbial communities, thereby improving overall functional stability and probiotic performance.

In the natural environment, *B. subtilis* biofilms are established within a diverse microbial community, where microbial metabolites such as toxins, nutrients, and signaling molecules collectively influence both microbial populations and biofilm structure.^13-15^ For example, *Bacillus cereus* and *Streptococcus mutans* have been shown to enhance *B. subtilis* biofilm formation.^16^ In contrast, *B. subtilis* metabolites can effectively eradicate established pathogenic bacterial populations.^17^ These reciprocal interactions may contribute to gut microbiome modulation by reinforcing beneficial microbial dynamics while suppressing pathogens. In addition, the natural coexistence of beneficial microorganisms within multispecies communities, such as *B. subtilis* biofilms, could provide an alternative strategy for designing mixed-species probiotic delivery formulations.^18^ It has been reported that co-culturing with *B. subtilis* biofilms improves the gastrointestinal stress tolerance of lactic acid bacteria.^19^ Environmental conditions are another important factor influencing *B. subtilis* biofilm communities, as *B. subtilis* develops stress-tolerant biofilms under elevated temperatures and alkaline pH.^20^ This observation is particularly interesting because unfavorable gastrointestinal conditions, such as alkaline pH, may trigger biofilm formation, thereby enhancing the survival and persistence of vegetative cells within the gastrointestinal tract, a feature generally lacking in free vegetative probiotic cells. Furthermore, the positive modulation of *B. subtilis* and its biofilms within the host and gut microbial community further supports their application as probiotics. For instance, *B. subtilis* strain DE111 improves gut bacterial diversity and increases beneficial bacterial populations in children.^21^ Host immune modulation is another key feature of microbial biofilms, as *B. subtilis* exopolysaccharides protect mice from *Citrobacter rodentium*-induced colitis by activating anti-inflammatory immune cells and suppressing excessive T-cell responses.^16^ ^,^ ^22^ Collectively, these distinctive properties of *B. subtilis* biofilms underscore their potential applications which extend beyond traditional spore- and vegetative cell-based formulations.

Here, we demonstrate that vegetative cells of *B. subtilis* exhibit poor survival under simulated gastrointestinal conditions, whereas a biofilm-overproducing *B. subtilis* strain shows enhanced resistance to gastrointestinal stress, particularly gastric juice, along with improved gut colonization and thermotolerance. These outcomes were compared with those of commercially available probiotics, *Lactobacillus rhamnosus* GG and the probiotic yeast *Saccharomyces boulardii*. Furthermore, we investigated the potential application of *B. subtilis* cell-free biofilms as an encapsulation material for probiotic formulation and delivery using beneficial bacterial strains, *Lactobacillus rhamnosus* GG, *Streptococcus thermophilus*, and *Escherichia coli* Nissle 1917. This biofilm-based encapsulated formulation improved freeze-processing stability and gastric stress resistance in the encapsulated vegetative probiotic strains.

## Results

### *B. subtilis* is more susceptible to gastric conditions compared to reference probiotics *Lactobacillus rhamnosus* GG and *Saccharomyces boulardii*

A defining characteristic of probiotic microorganisms is their ability to withstand the harsh conditions of the human gastrointestinal tract, including exposure to acidic gastric environments, bile salts, and digestive enzymes. However, these conditions can still be detrimental to many of the beneficial microorganisms delivered via probiotic formulations. To assess resilience under gastrointestinal conditions, we measured the survival of wild-type *B. subtilis*, strain NCIB 3610 following exposure to simulated gastric and bile juices that mimic conditions in the human gastrointestinal tract.^23^ The probiotic performance of *B. subtilis* was compared to that of commercially available reference probiotics, including the bacterium *Lactobacillus rhamnosus* GG and the yeast *Saccharomyces boulardii*.

**Figure 1.**
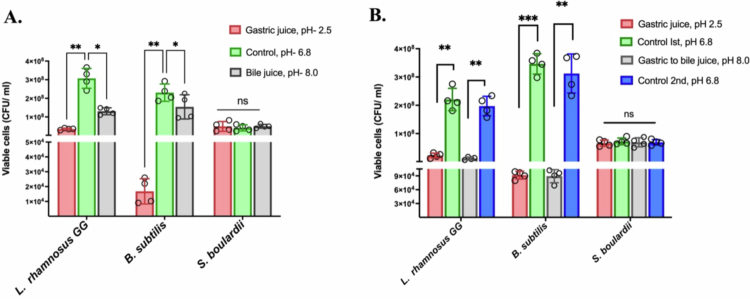
Gastrointestinal survivability of wild-type *B. subtilis* under *in vitro* sequential gastric-bile simulation. A total of 10⁸ cells/mL of the vegetative planktonic bacterium, *B. subtilis*, along with reference probiotics, *L. rhamnosus* GG and *S. boulardii,* are inoculated into synthetic digestive fluids consisting of gastric juice at pH 2.5 (red bars) and bile juice at pH 8.0 (gray bars), and incubated at 37 °C for 4 h. Control conditions consisted of electrolyte solutions without pepsin, bile, or pancreatic enzymes at pH 6.8 (green bars). Colony-forming units (CFUs) are enumerated using the viable count method (A). To mimic the sequential steps of the human digestive process *in vitro*, probiotics are first incubated in simulated gastric juice (pH 2.5) for 2 h (red bars). Cells are then harvested and transferred to bile juice (pH 8.0) for an additional 3 h (gray bars). CFUs are measured after exposure to each condition. The CFU count obtained from control samples incubated for 2 h at pH 6.8 (green bars; labeled control 1 st) served as a baseline for comparison with survival following gastric exposure. Subsequently, the control experimental cells that had undergone the initial gastric incubation are also used as the starting point for the bile-phase control and incubated for 3 h; their CFU values (blue bars; labeled control 2nd) are analyzed to assess the impact of bile on post-gastric survival (B).

Vegetative planktonic cultures of *B. subtilis*, *L. rhamnosus,* and *S. boulardii* were each inoculated at 10^8^ cells/mL, and survival was quantified by enumeration of colony-forming units (CFU) at 4 and 24 h post-inoculation. Exposure to simulated gastric juice (pH 2.5) resulted in a 99% reduction in viability of wild-type *B. subtilis* at 4 h post-inoculation compared to control conditions (pH 6.8 without pepsin). Under the same conditions, *L. rhamnosus* strain GG exhibited an 88% decrease in viability, whereas the viability of *S. boulardii* was not significantly affected at 4 h post-inoculation (Figure 1A). When exposed to simulated bile juice (pH 8.0), *B. subtilis* demonstrated a 67% reduction in viability at 4 h post-inoculation relative to control conditions (pH 6.8 without bile salts or pancreatic enzymes). *L. rhamnosus* GG showed a 57% decrease in viability, while *S. boulardii* again remained largely unaffected (Figure 1A).

To assess the effects of prolonged exposure to digestive conditions, microbial viability was measured 24 h post-inoculation in synthetic gastric and bile juices. *B. subtilis* and the reference probiotic yeast, *S. boulardii* did not exhibit further reductions in viability beyond those observed after 4 h of exposure. In contrast, the reference probiotic bacterium *L. rhamnosus* GG showed a significant additional decrease in viability following prolonged exposure to gastric juice (Supplementary Figure 1). Exposure to bile juice did not significantly affect microbial viability at 24 h post-treatment for any of the strains tested. Collectively, these results indicate that susceptibility to gastric juice is species dependent and generally more pronounced than susceptibility to bile juice. While *L*. *rhamnosus* GG exhibits partial resistance during short-term exposure, its survival decreases substantially with prolonged gastric exposure. Notably, wild-type vegetative *B. subtilis* cells are highly susceptible to gastric juice even following short-term exposure.

To further investigate microbial survival during digestion, we mimicked the sequential exposure encountered in the human gastrointestinal tract, in which probiotics are first exposed to gastric juice and subsequently to bile juice at 37 °C. Digestive transit time varies among individuals and is influenced by the type of food consumed; however, gastric retention is estimated to range from 15 minutes to 3 h, followed by transit through the small intestine over 2–5 h.^24^ To model these conditions experimentally, probiotic microbes were exposed to simulated gastric juice for 2 h, harvested, transferred to simulated bile juice, and incubated for an additional 3 h at 37 °C. Viability was assessed following each treatment. After 2 h of exposure to gastric juice at pH 2.5, *B. subtilis*, exhibited a 99% reduction in viability, while *L. rhamnosus* GG showed an 85% reduction relative to control conditions (pH 6.8). Subsequent exposure to simulated bile juice did not further compromise the viability of either strain. In contrast, the probiotic yeast, *S. boulardii* remained unaffected by both gastric and bile treatments (Figure 1B). These results indicate that gastric juice poses the greatest threat to survival of beneficial microbes such as *B. subtilis* in the gastrointestinal tract and highlight the need for protective strategies to enhance survival during gastric transit.

Next, we examined the effect of pH on microbial viability. The human digestive system encompasses a broad pH range, with stomach pH varying from approximately 1.5 to 5 during food consumption, and the intestinal lumen typically exhibits alkaline conditions.^25^ To investigate microbial survival along this pH gradient, we evaluated the viability of *B. subtilis* and compared it with the reference probiotics, *L. rhamnosus* GG and *S. boulardii*. Using standard growth media, Lysogeny Broth (LB), De Man-Rogosa-Sharpe (MRS) and Yeast Extract Peptone Dextrose (YEPD), we assessed viability across pH values ranging from 1.5 to 9.5 (in 1-unit intervals) at 37 °C for 4 h, relative to control conditions of pH 6.8. Under acidic conditions (pH ≤ 3.5), *B. subtilis* viability decreased by 99%, while *L. rhamnosus* GG showed a 56% reduction compared to the control. The probiotic yeast *S. boulardii* demonstrated greater tolerance to acidic conditions, with viability reductions of 10% and 48% at pH 3.5 and 2.5, respectively (Supplementary Table 1). Under alkaline conditions, *B. subtilis* viability decreased by 28% and 49% at pH 8.5 and 9.5, respectively, whereas *L. rhamnosus* GG and *S. boulardii* did not exhibit significant changes (Supplementary Table 1). Together, these findings suggest that probiotic microbes are generally more susceptible to acidic conditions (pH ≤ 3.5) than alkaline environments.

Finally, we investigated the effect of bile salts on microbial viability. Bile salts exert antimicrobial activity and play a key role in shaping the gut microbiome.^26^ In the human intestine, bile salt concentrations typically range from 3.87 to 10.34 mM (approximately 0.15% to 0.45%).^27^ We assessed microbial viability in the presence of ox-bile at concentrations ranging from 0.1% to 2%. *B. subtilis* exhibited a marked reduction in viability, with a 66% decrease observed at 0.1% bile and an approximately 99% reduction at concentrations of 0.3% or higher relative to the bile-free control. In comparison, *L. rhamnosus* GG showed sensitivity only at bile concentrations of 1% or greater, resulting in an 80-90% decrease in viability. Conversely, *S. boulardii* demonstrated high bile tolerance, with no significant reduction in viability across all tested concentrations (Supplementary Table 2). Together, these results reveal species-dependent differences in bile resistance. *S. boulardii* exhibits the greatest tolerance, *L. rhamnosus* GG shows reduced viability only at higher bile concentrations, and *B. subtilis* displays sensitivity even at lower bile levels, although its survival remains within physiologically relevant ranges.

### *B. subtilis* shows limited colonization in *C. elegans* gut

**Figure 2.**
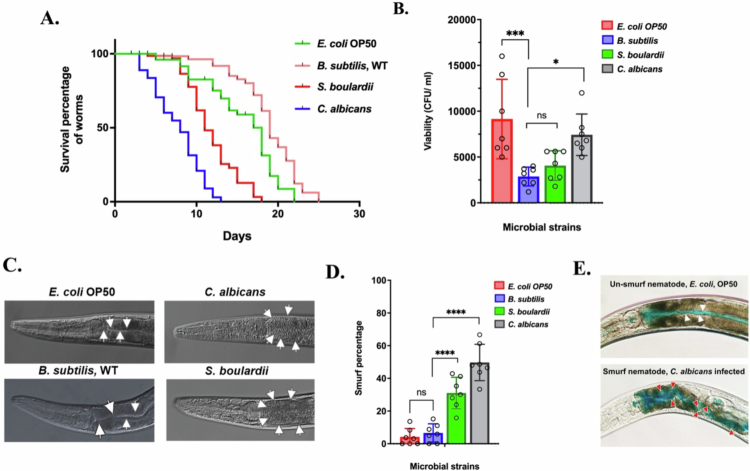
Colonization and nonpathogenic profile of *B. subtilis* in *C. elegans*. The effects of *B. subtilis* on the survival and longevity of *C. elegans* is assessed by rearing worms on *B. subtilis* lawns prepared on NGM plates. Live and dead worms are counted daily until all worms on plate had died. *E. coli* OP50 is used as the standard diet, and *S. boulardii* is used as a reference probiotic strain. Pathogenic effects are evaluated using worms infected with *C. albicans*, which served as a positive control (A). The colonization ability of *B. subtilis* and other microbes is evaluated by exposing synchronized worms to microbial lawns. After overnight incubation, worms are thoroughly washed to remove microbes adhering to the cuticle, and internal colonization is quantified by plating homogenized worm suspensions (B). Microscopic images of worms colonized with *B. subtilis*, *E. coli* OP50, *S. boulardii*, and *C. albicans* are captured from the anterior region of the worm (C). A Smurf assay is used to assess epithelial damage in the nematode gut. Worms exposed overnight to *B. subtilis*, *E. coli* OP50, *S. boulardii*, or *C. albicans* are stained with 5% erioglaucine disodium salt. The percentage of worms exhibiting dye leakage into the body cavity is quantified relative to the total number of worms examined microscopically (D). A representative micrograph showing intact (non-Smurf) and damaged (Smurf) intestinal lumens is presented to illustrate gut integrity in the nematode (E).

Next, we assessed the ability of wild-type *B. subtilis* to colonize the intestine of *C. elegans*. *C. elegans* has been widely used as a model host for preliminary investigation of host-microbe interactions since it shares physiologically relevant features of the gut epithelial barrier and innate immunity with humans.^28^ Here, we examined the effects of *B. subtilis* exposure on host survival by monitoring nematode lifespan and comparing it with *S. boulardii,* a known probiotic.^29^ Worms reared on *B. subtilis* exhibited increased longevity compared to those fed the standard laboratory food *Escherichia coli* OP50, with median survival times of 19 and 18 d, respectively. In contrast, worms reared on *S. boulardii* had a median survival of 11 d, while those fed the pathogenic fungus *C. albicans* died within 8 d. These results indicate that *B. subtilis* enhances nematode lifespan relative to the standard lab chow of *E. coli* OP50 (Figure 2A).

Next, we assessed intestinal microbial accumulation as described previously.^30^ Synchronized worms were exposed to microbial lawns overnight, washed extensively to remove external microbes, and homogenized to quantify internal colonization by plating. *B. subtilis* exhibited significantly reduced intestinal colonization compared to both *C. albicans* and *E. coli* OP50, which served as the pathogenic positive control and standard diet, respectively. In contrast, no statistically significant difference in gut colonization was observed between *B. subtilis* and the reference probiotic *S. boulardii* (Figure 2B). Notably, nematodes colonized by *C. albicans* or *S. boulardii* displayed pronounced intestinal distension, a phenotype absent in worms fed *B. subtilis* or *E. coli* OP50 (Figure 2C). To determine whether intestinal distension was associated with compromised epithelial barrier function, we performed a dye permeability assay, in which epithelial disruption is indicated by dye diffusion into the body cavity. Worms reared on *B. subtilis* maintained gut epithelial integrity comparable to those fed the *E. coli* OP50 diet. In contrast, 49% of worms colonized by the pathogen *C. albicans* exhibited a leaky gut phenotype, while *S. boulardii* exposure resulted in moderate epithelial compromise, with 30% of worms displaying dye leakage (Figure 2D and E). Together, these findings demonstrate that although wild-type *B. subtilis* exhibits limited intestinal colonization relative to some reference microbial strains, it does not display pathogenicity toward *C. elegans*. Instead, *B. subtilis* engages with the host in a manner consistent with a beneficial association.

### *B. subtilis* strains that overproduce biofilms survive harsh gastrointestinal conditions

**Figure 3.**
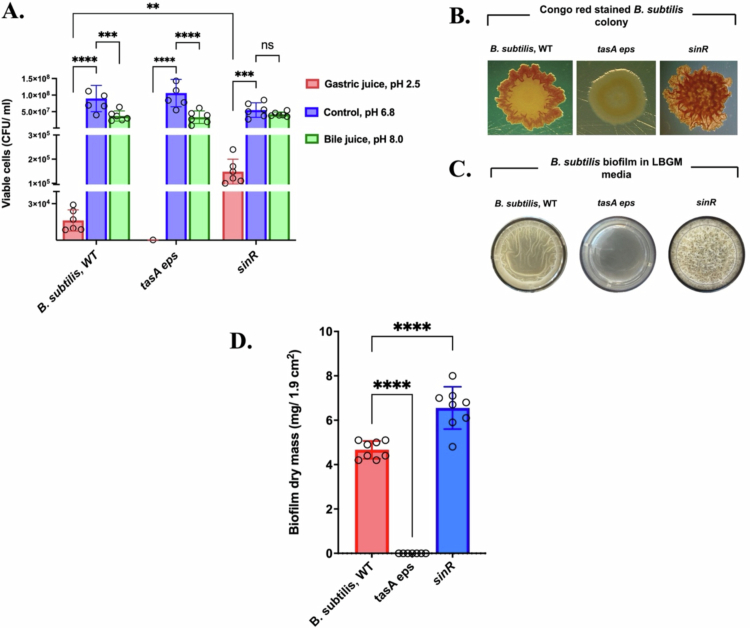
Biofilm-overproducing *B. subtilis sinR* enhances tolerance to gastric fluid. Wild-type *B. subtilis*, the biofilm-overproducing mutant *sinR*, and the biofilm-deficient mutant *tasA eps* are exposed to simulated gastric (red bars) and bile (green bars) fluids and incubated at 37 °C for 4 h with mild shaking. Control conditions consisted of electrolyte solutions without pepsin, bile, or pancreatic enzymes and are maintained at pH 6.8 (blue bars). Viability is assessed by serial dilution, and results are expressed as CFU/mL (A). Biofilm production is visualized using Congo red staining. Bacterial strains are cultured on LB agar plates supplemented with Congo red and incubated at 37 °C for 48 h. Colony morphology and dye retention are imaged using a stereo microscope (B). Biofilms of wild-type *B. subtilis*, *sinR,* and *tasA eps* mutants are grown in LBGM medium in 24-well plates and incubated under static conditions at 37 °C for 24 h(C), then biofilms are harvested, dried at 40 °C for 48 h, and the weight is measured (D).

To assess the ability of *B. subtilis* to persist in the host, we evaluated its ability to survive gastrointestinal transit and colonize the host. Tolerance to gastric acidity is a prerequisite for survival and colonization, which in turn supports functions such as strengthening the gut barrier and modulating host immune responses. Our findings indicate that wild-type *B. subtilis* exhibits limited intestinal colonization and modest tolerance to gastric juice, suggesting reduced survival during gastrointestinal transit. Microbial biofilms are well documented for their enhanced resistance to environmental stressors, including acidity and chemical challenges.^31^ We therefore hypothesized that *B. subtilis* strains capable of robust biofilm formation would exhibit increased tolerance to gastric and bile stress, thereby improving survival during gastrointestinal transit. To test this hypothesis, we examined a biofilm-overproducing *B. subtilis* mutant, *sinR,* and a biofilm-deficient double mutant *tasA eps* and the isogenic wild-type strain. These strains were exposed to simulated gastric and bile juices for 4 h. The biofilm-overproducing *sinR* mutant exhibited markedly enhanced survival under acidic conditions, with approximately 95% higher viability than the wild-type strain (Figure 3A). In contrast, the *tasA eps* mutant, which lacks key structural components of the biofilm matrix, showed increased susceptibility to gastric stress and a substantial loss of viability. Exposure to bile juice did not result in significant differences in viability among the three strains (Figure 3A). These results indicate that enhanced biofilm formation protects *B. subtilis* specifically in acidic gastric environments.

To further support this observation, we examined *B. subtilis* mutants that produce partial or incomplete biofilms (Supplementary Table 3), representing intermediate phenotypes between the biofilm-defective, *tasA eps* strain and the wild-type strain, which forms a mature biofilm. These intermediate strains also failed to survive gastric conditions (Supplementary Figure 2). Collectively, these results demonstrate that gastric survival correlates with robust biofilm overproduction rather than the mere ability to form partial or immature biofilms. The *sinR* mutant exceeds this threshold, indicating that substantially elevated biofilm production confers a survival advantage during gastric transit.

To validate biofilm-forming capacity, we assessed extracellular matrix (ECM) production using Congo red staining. Congo red binds ECM polysaccharides and provides a qualitative measure of biofilm formation. The biofilm-overproducing *sinR* mutant exhibited the strongest Congo red binding, consistent with increased ECM production, whereas the wild-type strain showed moderate staining. In contrast, the biofilm-deficient *tasA eps* mutant displayed minimal Congo red absorption, reflecting impaired ECM synthesis (Figure 3B). These qualitative observations were further substantiated by quantitative measurements of biofilm biomass. When cultured under biofilm-promoting LBGM (Lysogeny broth supplemented with glycerol and manganese) media, the *sinR* mutant formed a biofilm weighing 6.55 mg over a 1.9 cm² surface area, representing a 40.6% increase in biofilm mass compared to the wild-type strain. The *tasA eps* failed to form detectable biofilm (Figure 3C and D). We also evaluated biofilm production in MSgg medium; however, the pellicles formed were structurally fragile and difficult to harvest.

Next, we assessed the effect of pH on microbial viability by culturing the *sinR* and *tasA eps* mutants and wild-type strain in LB medium adjusted to pH values ranging from 1.5 to 9.0. Exposure to acidic conditions (pH ≤ 3.5) significantly compromised viability across all strains, with a clear correlation between biofilm-forming capacity and pH sensitivity. The biofilm-deficient *tasA eps* mutant exhibited complete loss of viability at pH ≤ 3.5. The wild-type strain showed approximately a 99% reduction in viability relative to the pH 6.8 control. In contrast, the *sinR* mutant demonstrated enhanced acid tolerance, with 71% and 97% greater survival at pH 2.5 and pH 3.5, respectively, compared to the wild type strain (Supplementary Figure 3). Alkaline conditions (pH 8.5 and 9.0) did not significantly affect viability in any of the strains, indicating tolerance to alkaline environments regardless of biofilm-forming capacity. These findings further bolster the conclusion that a robust biofilm protects *B. subtilis* from gastric stress.

Finally, we evaluated the effect of bile on *B. subtilis* survival by culturing in LB medium supplemented with increasing concentrations of ox-bile (0.1%, 0.3%, 0.6%, 1%, and 2%). All strains exhibited a marked reduction in viability at bile concentrations of ≥0.3% relative to bile-free controls. No significant differences in bile tolerance were observed among strains, regardless of their biofilm-forming ability (Supplementary Figure 4). These results indicate that while biofilm overproduction enhances survival of *B. subtilis* under acidic conditions, it does not confer protection against bile-mediated stress, suggesting that biofilms provide selective protection specifically against gastric acidity.

### Biofilm overproducing *B. subtilis* sinR enhances adhesion and intestinal colonization

**Figure 4.**
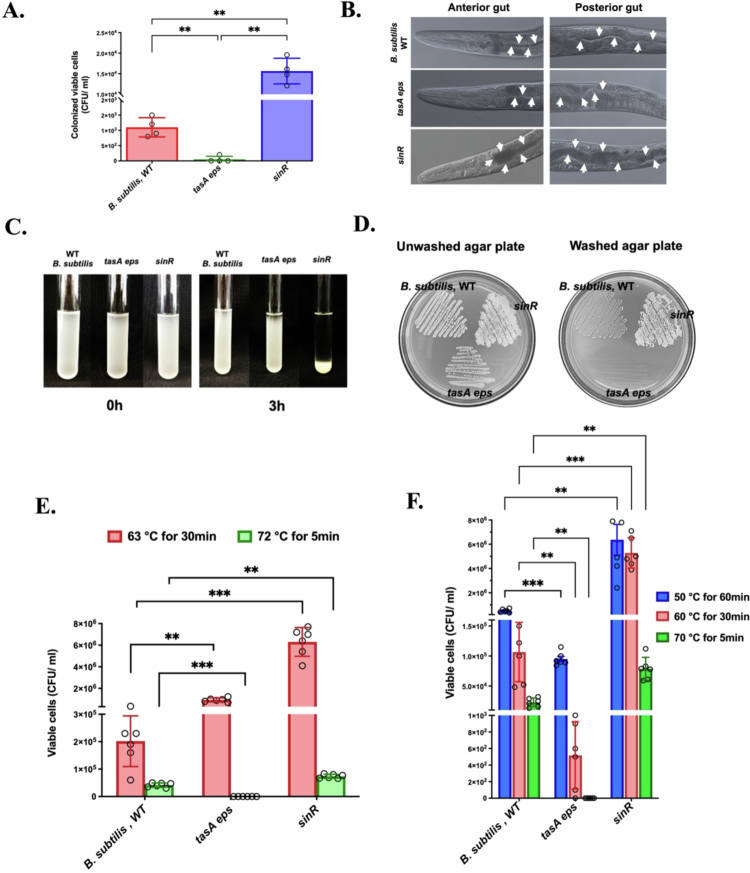
Biofilm-overproducing strain *B. subtilis sin* *R* promotes adhesion, colonization, and thermal tolerance. Nematodes are exposed overnight to wild-type *B. subtilis*, *sinR*, and *tasA eps* strains. Following exposure, worms are collected, homogenized, and serially diluted. The suspensions are then plated to quantify bacterial colonization, and expressed as CFU/mL (A). Hoffman modulation contrast (HMC) microscopy is performed to visualize bacterial localization within the anterior and posterior intestinal regions of the nematode gut (B). Bacterial flocculation is assessed by suspending cells at an OD of 1.0 in PBS and allowing them to settle undisturbed at room temperature for 3 h (C). Surface adhesion properties are evaluated by streaking each strain on LB agar plates followed by incubation at 37 °C for 24 h. Plate images are captured before and after gentle washing to assess retention of bacterial colonies (D). Pasteurization tolerance is tested by subjecting 10⁸ cells/mL of each strain to 63 °C for 30 min (red bars) and 72 °C for 5 min (green bars). Samples are immediately cooled on ice for 5  min, and post-treatment viability is determined by CFU enumeration (E). Thermal tolerance is evaluated by exposing bacterial suspensions (10⁸ cells/mL) to 50 °C for 60 min (blue bars), 60 °C for 30 min (red bars), and 70 °C for 5 min (green bars). After cooling to room temperature, cell viability was quantified by plating appropriate dilutions and counting CFUs (F).

To investigate the role of biofilm formation in intestinal colonization *in vivo*, *C. elegans* were exposed to bacterial lawns, and colonization efficiency was quantified using CFU enumeration of ingested bacteria. The biofilm-overproducing *B. subtilis sinR* mutant exhibited a 92% increase in intestinal colonization compared to the wild-type strain. In contrast, the biofilm-deficient *tasA eps* mutant showed an 85% reduction relative to the wild-type and a 96% reduction relative to *sinR* (Figure 4A and B). To assess the physiological consequences of enhanced colonization, lifespan assays were performed in *C. elegans* fed wild-type *B. subtilis*, *sinR,* or the *tasA eps* mutant. No significant differences in survival were observed between worms fed the standard *E. coli* OP50 diet and those exposed to the *sinR* strain, indicating that increased colonization by *sinR* does not adversely affect host longevity. In contrast, worms fed with wild-type *B. subtilis* or the *tasA eps* mutant exhibited a modest yet statistically significant increase in lifespan relative to *sinR-* and *E. coli* OP50-fed controls (Supplementary Figure 5). These findings suggest that enhanced biofilm-mediated colonization alone is insufficient to modulate lifespan, and that host fitness may instead be influenced by strain-specific traits or metabolite-mediated effects on host physiology.^32^ Nonetheless, enhanced gut colonization by gut commensals is generally considered advantageous, as stable resident microbiota can promote epithelial integrity, inhibit pathogenic colonization through microbial antagonism, and secrete bioactive metabolites that enhance digestion as well as strengthen epithelial defenses, thereby supporting host physiological fitness.

In microbial communities, flocculation, biofilm formation, and cell adhesion are often co-regulated processes that collectively enhance colonization and survival under diverse environmental conditions.^33^ Similar to biofilms, flocs can provide a protective microenvironment that enhances tolerance to stress. To evaluate flocculation as an *in vitro* proxy for biofilm-associated colonization *in vivo*, cultures of each strain were normalized to an OD_600_ of 1.0 and incubated at room temperature for 3 h. Flocculation was assayed visually. The biofilm-overproducing *sinR* mutant exhibited rapid sedimentation compared to the wild-type strain, indicative of a strong flocculation phenotype. In contrast, the biofilm-deficient *tasA eps* mutant displayed flocculation comparable to that of the wild-type (Figure 4C), suggesting that the loss of biofilm matrix components does not substantially impair flocculation, whereas enhanced biofilm production markedly promotes this behavior. Because flocculation and adhesion phenotypes are often correlated,^33^ we next assessed surface adhesion by growing bacterial patches on LB agar plates for 24 h, followed by gently washing to evaluate bacterial attachment. The *sinR* mutant remained strongly adherent to the agar surface, whereas wild-type *B. subtilis* exhibited moderate adhesion and the biofilm-deficient *tasA eps* mutant failed to adhere (Figure 4D). No agar invasion was observed for any of the strains, a phenotype often associated with virulence. This observation is consistent with *C. elegans* survival assays, in which no reduction in lifespan was detected relative to *E. coli* OP50-fed controls, further supporting the nonpathogenic nature of these strains. Collectively, these results demonstrate a functional relationship among biofilm formation, flocculation, and surface adhesion in *B. subtilis*, highlighting interconnected regulatory mechanisms that enhance colonization potential. Notably, these phenotypes were most pronounced in the biofilm-overproducing *sinR* mutant strain, underscoring the close association between biofilm overproduction and enhanced host-associated intestinal colonization.

### Biofilm-overproducing *B. subtilis*, *sinR* exhibits enhanced thermotolerance

Having demonstrated that biofilm overproduction in *B. subtilis* enhances adhesion and resistance to gastrointestinal stress, we next examined whether this phenotype also confers advantages under industrial processing conditions relevant to microbial formulation. Common preservation and processing methods, such as spray drying, fluidized-bed drying, vacuum drying, and lyophilization, subject microbial cells to extreme temperature and pressure changes that can significantly reduce viability.^5^ Similarly, pasteurization, although effective for food safety, can adversely affect beneficial microbial populations. To evaluate resistance to pasteurization-induced thermal stress, wild-type *B. subtilis*, the biofilm-overproducing mutant *sinR,* and the biofilm-deficient *tasA eps* mutant were exposed to two standard pasteurization conditions: 63 °C for 30  min and 72 °C for 5 min, followed by rapid cooling on ice. As expected, heat treatment reduced viability across all strains. However, the *sinR* mutant exhibited significantly greater survival, with 96% and 56% higher viable cell counts than its wild-type counterpart following exposure to 63 °C and 72 °C, respectively (Figure 4E). Compared with the *tasA eps* mutant, *sinR* showed an 85% increase in viability at 63 °C, whereas *tasA eps* failed to survive exposure to 72 °C (Figure 4E).

To determine whether this enhanced survival extended beyond pasteurization conditions, general thermal tolerance was subsequently assayed by exposing cells to 50 °C for 60 min, 60 °C for 30 min, or 70 °C for 5 min, followed by cooling at room temperature. Exposure to 50 °C had minimal effects on all strains. At higher temperatures, the *sinR* mutant consistently displayed enhanced resilience compared with the wild-type strain. In contrast, the *tasA eps* mutant was unable to survive treatment at 60°C or 70 °C (Figure 4F). These findings suggest that robust biofilm formation is associated with enhanced thermal tolerance and may improve suitability for heat-intensive processing methods such as spray drying, fluidized-bed drying, and vacuum drying.

Lyophilization is widely used for the long-term preservation of microbial formulations due to its ability to maintain stability without refrigeration. Despite its advantages, freeze-drying can impose multiple stresses, including dehydration, membrane damage, and oxidative stress. To assess the impact of lyophilization, the cell suspensions prepared in 10% (w/v) maltodextrin were subjected to freeze-drying. No significant differences in post-lyophilization viability were observed among the wild-type *B. subtilis*, *sinR*, and *tasA eps* strains, indicating that lyophilization does not differentially affect survival, regardless of biofilm-forming capacity (data not shown).

### Bioencapsulated with *B. subtilis* biofilm enhances the gastric survival of direct-fed microbials

Encapsulation is a widely used strategy to protect beneficial microorganisms during processing, storage, and passage through the harsh conditions of the gastrointestinal tract. Encapsulation typically involves coating cells with protective biomaterials that help preserve viability and functionality. We hypothesized that the extracellular biofilm matrix produced by *B. subtilis* could serve as a natural encapsulation material, thereby enhancing the resistance of encapsulated vegetative microbial cells to gastric stress and improving their delivery to the host.

**Figure 5.**
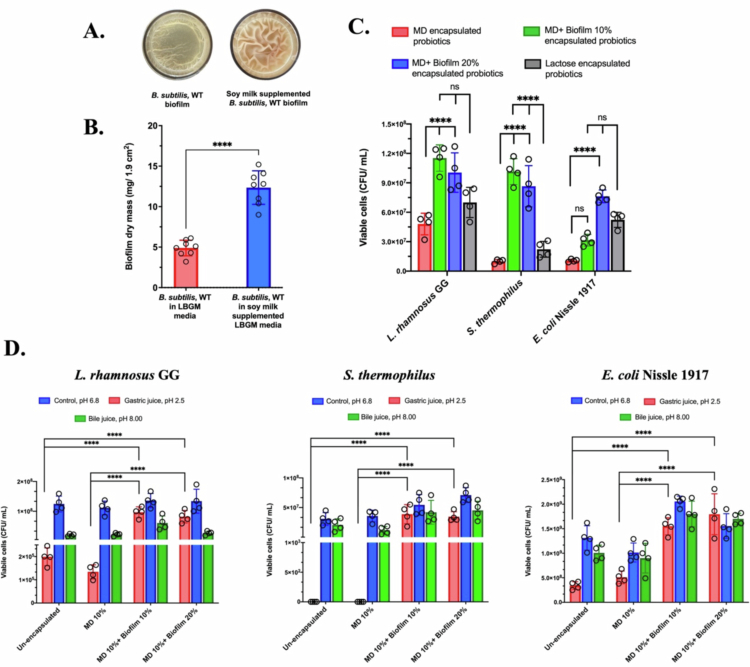
Cell-free wild-type *B. subtilis* biofilm-encapsulated probiotics exhibit improved viability during lyophilization and enhanced survival in gastric juice. Biofilm formation by wild-type *B. subtilis* is enhanced by supplementing LBGM medium with 50% soy milk (A, B). Biofilms are then harvested and heat-treated at 121 °C for 20 min. Heat-treated *B. subtilis* biofilms at concentrations of 10% w/v (green bars) and 20% w/v (blue bars) are blended with 10% maltodextrin (MD) and used as encapsulation matrices for the vegetative probiotic strains *L. rhamnosus* GG, *Streptococcus thermophilus*, and *E. coli* Nissle 1917. Encapsulation is performed by lyophilization for 24–30 h. Post-lyophilization viability of the encapsulated probiotics is assessed by serial dilution and expressed as CFU/mL. A 10% maltodextrin formulation served as the untreated control (red bars), while 10% lactose is used as the reference control (gray bars) (C). Next, the encapsulated formulations of *L. rhamnosus* GG, *Streptococcus thermophilus*, and *E. coli* Nissle 1917 are exposed to simulated gastric (red bars) and bile juices (green bars) for 4 h, after which viability is determined and expressed as CFU/mL. Electrolyte solutions lacking digestive enzymes and bile components at pH 6.8 are used as baseline control conditions in this experiment (blue bars). Nonencapsulated probiotics and probiotics encapsulated with 10% maltodextrin (10% MD) are used as untreated and vehicle (matrix) control, respectively (D).

To generate sufficient biofilm material for encapsulation, we first optimized biofilm production by supplementing the growth medium with soy milk. Soy milk supplementation resulted in an approximately 153% increase in biofilm biomass compared with LBGM medium alone (Figure 5A and B, Supplementary Figure 6). Because *B. subtilis* biofilms can contain substantial numbers of spores, posing a potential contamination risk in encapsulated formulations, the harvested biofilms were heat-inactivated by autoclaving at 121 °C for 20 min. Given the importance of biosafety in encapsulation materials, we next assessed the cytotoxicity of the heat-treated biofilm. Lyophilized biofilm powder was applied to Caco-2 cells at concentrations of 1, 10, and 50 mg/mL for 2 and 6 h, and cytotoxicity was evaluated using an MTT assay. No detectable cytotoxic effects were observed, indicating that the heat-inactivated biofilm matrix is non-toxic and suitable for downstream applications (Supplementary Figure 7).

The heat-inactivated biofilm was subsequently incorporated into encapsulation matrix formulations consisting of 10% (w/v) maltodextrin supplemented with either 10% or 20% (w/v) biofilm. These matrices were used to encapsulate *L. rhamnosus* GG, *Streptococcus thermophilus*, and *Escherichia coli* Nissle 1917, each standardized to an initial density of 10⁸ CFU/mL prior to lyophilization. Maltodextrin, a commonly used and cost-effective carrier, was included to reduce the stickiness and hygroscopicity of the final product. A 10% lactose matrix was included as a reference, as lactose is widely used as a cryoprotectant and lyoprotectant in freeze-dried microbial formulations. Biofilm-supplemented matrices significantly improved survival during lyophilization. Compared with maltodextrin alone, viability increased by approximately 1–2 fold for *L. rhamnosus* GG, 7–9 fold for *S. thermophilus*, and 1–6 fold for *E. coli* Nissle 1917. Viability in the biofilm-encapsulated formulations was comparable to, or higher than, that achieved with lactose-encapsulated strains (Figure 5C). These findings suggest that encapsulation with the extracellular matrix of *B. subtilis* biofilm provides effective protection during freeze-drying.

We next assessed whether this bio-encapsulation formulation enhanced survival under simulated gastrointestinal conditions. Encapsulated microbial cells were exposed to synthetic gastric and bile juices for 4 h. Biofilm-supplemented formulations significantly improved survival in gastric juice. Relative to vehicle control, maltodextrin-only formulations, and non-encapsulated vegetative planktonic cells, survival increased approximately 99% for *L. rhamnosus* GG, 70-79% for *E. coli* Nissle 1917, and approximately 100% for *S. thermophilus*. In contrast, no substantial differences in survival were observed following exposure to bile across treatment groups (Figure 5D). These results indicate that encapsulation by the extracellular biofilm matrix of *B. subtilis* specifically protects against gastric, but not bile-mediated, stress.

We hypothesized that this protective effect is primarily driven by the biofilm's ability to buffer acidic environments. To test this hypothesis, 100 ± 15 mg of lyophilized powder containing biofilm-supplemented maltodextrin, biofilm alone, or maltodextrin alone were added to varying volumes (2, 3, 5, and 10 mL) of synthetic gastric juice (initial pH 2.5 ± 0.1). Biofilm-containing formulations effectively increased the pH of the gastric juice, raising it to 4.3–5.0, 3.8–4.6, 3.9–4.3, and 3.0–3.4 in 2, 3, 5, and 10 mL volumes, respectively (Table 1). In contrast, maltodextrin alone had minimal impact on pH, indicating that the buffering capacity is attributable to the biofilm matrix.

**Table 1.** pH-buffering capacity of *B. subtilis* biofilm.

	Volume of gastric juice added to the lyophilized sample				
Lyophilized formulations	2 mL	3 mL	5 mL	10 mL	Altered pH of gastric juice at pH
Maltodextrin (10%)	2.69 ± 0.05	2.66 ± 0.01	2.62 ± 0.02	2.60 ± 0.02	2.50 ± 0.1
Biofilm (10%)	4.31 ± 0.07	3.89 ± 0.18	3.90 ± 0.14	3.02 ± 0.20	
Biofilm (20%)	4.79 ± 0.05	4.33 ± 0.15	4.04 ± 0.25	3.26 ± 0.06	
Maltodextrin (10%) + Biofilm (10%)	4.46 ± 0.07	3.92 ± 0.10	3.51 ± 0.14	2.96 ± 0.11	
Maltodextrin (10%) + Biofilm (20%)	5.11 ± 0.23	4.62 ± 0.08	4.31 ± 0.09	3.45 ± 0.07	

Next, to determine whether the bioencapsulation matrix also confers direct biochemical protection independent of pH buffering, we exposed bio-encapsulated formulations to gastric juice, allowed to increase the pH, and then readjusted the pH to 2.5 ± 0.1 before incubation at 37 °C for 4 h. Under these conditions, microbial survival was not improved (data not shown). These results indicate that the primary protective mechanism of the *B. subtilis* biofilm matrix is environmental pH neutralization rather than the direct induction of acid-resistance pathways or biochemical modification of the encapsulated vegetative probiotic cells. These findings demonstrate that the *B. subtilis* biofilm matrix enhances microbial survival under gastric stress by buffering acidic conditions, supporting its potential as a natural and effective encapsulation material for acid-sensitive live biotherapeutics and direct-fed microbials.

## Discussion

Several strategies have been explored to enhance the functional performance of live biotherapeutics and direct-fed microbials, including the selection of stress-resistant strains, advanced encapsulation technologies, adaptive evolution approaches, and the use of gut-adapted microorganisms. In this study, we show that vegetative planktonic wild-type *B. subtilis* cells are highly susceptible to gastric acidity (pH ≤ 3.5), a major barrier to survival during gastrointestinal transit. In contrast, a biofilm-overproducing *B. subtilis* strain exhibited markedly enhanced survival under simulated gastric conditions. Based on this observation, we further demonstrate that cell-free biofilms derived from *B. subtilis* can be used as a bioencapsulation material. This represents a promising strategy for overcoming both functional and technological limitations associated with the formulation of live biotherapeutics. *B. subtilis* biofilms consist of an extracellular matrix composed of polysaccharides, protein fibers, and extracellular DNA (eDNA) that encases bacterial cells within a structured multicellular community. This complex architecture provides protection against diverse environmental stresses, including acidic pH and elevated temperatures, by creating a buffered microenvironment and limiting the direct exposure of individual cells to hostile conditions.

To dissect the role of biofilm formation in stress tolerance, specifically during gastric transit and downstream processing of live biotherapeutics, we examined two well-characterized *B. subtilis* mutants. The biofilm-deficient *tasA eps* mutant, which lacks essential extracellular matrix components and amyloid TasA, a major protein in the extracellular matrix, thereby disrupting multicellular biofilm formation^34^ ^,^ ^35^, failed to survive exposure to gastric juice and elevated temperatures. *SinR* is a transcriptional regulator controlled by SinI, SlrA, and SlrR, which together form a molecular switch that determines SinR-mediated repression of biofilm formation through regulation of extracellular matrix-associated operons.^36^ ^,^ ^37^ Therefore, deletion of *sinR* results in robust biofilm formation and, in this study, was associated with substantially improved tolerance to gastric acidity. In agreement with prior studies, we demonstrate that biofilm overproduction not only enhances additional functional traits, such as adhesion and flocculation, but also improves gastric survival, gut colonization, and thermotolerance during processing. These findings suggest that biofilm-rich beneficial microbial strains simultaneously enhance multiple beneficial attributes rather than improving a single isolated trait, thereby distinguishing this strategy from conventional probiotic approaches that primarily rely on spore-based and vegetative cells.

It is well established that biofilms formed by *B. subtilis* protect nematodes from heat and oxidative stress and can also extend worm lifespan.^38-40^ In our study, wild-type *B. subtilis* enhanced worm longevity more effectively than *E. coli* OP50 and the probiotic yeast, *S. boulardii*. However, we did not observe increased longevity in the biofilm-overproducing *sinR* mutant strain. We speculate that the biofilm produced by wild-type *B. subtilis* represents an optimal state for promoting beneficial effects in *C. elegans*, whereas the enhanced biofilm-forming ability of *sinR* mutant may increase bacterial resistance to worm digestion and limit nutrient availability. This may reflect a limitation of the *C. elegans* model in capturing host–microbe interactions relevant to higher organisms, and further studies in mammalian models will be required to validate these observations.

Enhanced biofilm formation was associated with increased surface adhesion and flocculation, highlighting the coordinated regulation of these phenotypes in biofilm-overproducing microbes such as the *sinR* mutant. Similar correlations between flocculation and adhesion have been reported in other microbial systems.^33^ Flocculation promotes microbial aggregation into dense, multilayered structures that provide physical protection and create a stable microenvironment that shields inner cells from external stresses. Such aggregation may also facilitate transient colonization by promoting stable contact with intestinal epithelial surfaces. Consistent with this idea, previous studies have demonstrated that disruption of flocculation in pathogens such as *Candida glabrata* reduces their ability to adhere to abiotic surfaces,^41^ underscoring the importance of flocculation in microbial adhesion.

Biofilm formation in *B. subtilis* can be induced by specific environmental cues, including organic acids, glycerol, and metal ions.^42^ In this study, we enhanced biofilm formation by supplementing the growth media with soy milk and subsequently utilized the resulting biofilm as a matrix material for microbial encapsulation. Prior studies have shown that plant-derived polysaccharides and resistant starch fibers can stimulate *B. subtilis* biofilm formation,^43^ ^,^ ^44^ suggesting that polysaccharide components of soy milk likely contribute to this induction.

Notably, encapsulation using these biofilm matrices significantly enhanced the survival of severely acid-sensitive strains such as *S. thermophilus*, as this protective effect was associated with effective neutralization of gastric acidity. *B. subtilis* biofilms can regulate extracellular pH toward neutrality through balanced acetate and acetoin biosynthesis, which may provide a mechanistic explanation for the enhanced survival of biofilm-overproducing bacteria under simulated gastric conditions.^45^ However, because heat-inactivated biofilms were used as the encapsulation matrix in this study, the observed protection against gastric acidity is unlikely to result from active metabolic processes. Instead, structural components of the biofilm extracellular matrix, including polysaccharides, extracellular DNA (eDNA), and inorganic constituents such as metal ions and minerals,^16^ may contribute to buffering acidic conditions and reducing acid-induced cellular damage. It is notable that neither the biofilm-overproducing strain nor biofilm-based encapsulation conferred additional protection against bile-induced stress, consistent with the detergent-like action of bile salts, which disrupt lipid membranes and denature proteins through mechanisms that cannot be mitigated by pH buffering alone.

As a future perspective, the incorporation of biofilm-derived materials into encapsulation matrices may influence the broader gut microbial ecosystem. For instance, exopolysaccharides produced by *Ligilactobacillus salivarius* have been shown to beneficially modulate gut microbiota composition in poultry.^46^ These findings collectively raise the possibility that encapsulated *B. subtilis* biofilms or purified biofilm components may possess prebiotic-like functions. Investigating their interactions with the resident gut microbiota and associated microbial metabolites represents an important direction for future research. In addition, our biofilm-based probiotic encapsulation strategy demonstrated promising protective and functional properties for probiotics, however, comprehensive techno-economic analyses and pilot-scale validation will be necessary to determine its commercial applicability within the food and bioprocess industries.

In summary, our study demonstrates that vegetative planktonic wild-type *B. subtilis* cells are highly vulnerable to gastric stress. In contrast, biofilm-overproducing *B. subtilis* strains exhibit enhanced tolerance to gastric acidity, improved adhesion and colonization of the *C. elegans* gut, and increased thermotolerance. While beneficial microbes are traditionally delivered as vegetative cells, our findings indicate that biofilm-associated states provide intrinsic protection against both gastrointestinal and bioprocessing-related stresses. Additionally, biofilm-based encapsulation offers a complementary strategy to enhance the survival of acid-sensitive probiotics. Together, these approaches represent a biologically grounded alternative to conventional delivery methods, analogous in function to spore-based formulations, and highlight the potential of biofilm-associated systems for advancing the formulation of sensitive live biotherapeutics and direct-fed microbials.

## Methodology

### Microbial strains and culturing conditions

*B. subtilis* strain NCIB 3610 and its mutants(*sinR* and *tasA eps*), and probiotic bacterium, *E. coli* strain Nissle 1917 were cultured overnight in LB broth at 37 °C*. L. rhamnosus GG* and *S. thermophilus* were grown in MRS medium, while the yeasts *S. boulardii* and *C. albicans* were cultivated overnight in YEPD medium at 30 °C. Following incubation, all bacterial and yeast cultures were washed three times with PBS (pH 7.4), and the harvested cells were used for subsequent experiments. *B. subtilis* biofilm was developed in biofilm-promoting medium LBGM (Lysogeny broth supplemented with 1% glycerol and 0.1 mM MnSO₄^47^).

### Simulated gastrointestinal tolerance assay

An electrolyte solution was prepared by dissolving potassium phosphate monobasic (0.6 g/L), magnesium chloride (0.1525 g/L), sodium chloride (2.8559 g/L), calcium chloride (0.2646 g/L), and potassium chloride (0.8647 g/L) in Milli-Q water. Glucose (0.3500 g/L) and individual essential amino acids (0.020 g/L each) were subsequently added to the solution. This electrolyte composition was partially adapted from Stefaniak et al., 2010.^48^

To simulate gastric juice, pepsin was added to the electrolyte-glucose-amino acid solution at a concentration of 0.7 g/L, and the pH was adjusted to 2.5 using 6 N hydrochloric acid. Simulated bile juice was prepared by supplementing the same electrolyte-glucose-amino acid solution with pancreatin (0.750 g/L) and ox bile (3.0 g/L), followed by pH adjustment to 8.0 using sodium hydroxide.^29^

A control solution was prepared using the same base electrolyte-glucose-amino acid composition, but without the addition of digestive enzymes (pepsin or pancreatin) or bile. The pH of the control was maintained at 6.8.

Bacterial and/or yeast cultures were inoculated individually into the respective media at 10⁸ cells/mL and incubated at 37 °C for 4 and 24 h under mild shaking. Postincubation samples were serially diluted and plated on appropriate agar media: LB for *B. subtilis* and its mutants, MRS for *L. rhamnosus* GG, and YEPD for *S. boulardii*. Results were expressed as CFU/mL.

To mimic the digestive process of the human gastrointestinal tract, the test probiotic *B. subtilis* and reference strains *L. rhamnosus* GG and *S. boulardii* were first exposed to simulated gastric juice for 2 h at 37 °C under mild agitation. Following gastric exposure, the cells were harvested by centrifugation and subsequently incubated in simulated bile juice for 3 h at 37 °C. Cell viability was assessed after each treatment stage (gastric and bile exposure) by serial dilution and plating, and the results were expressed as CFU/mL.

In the control group, cells were incubated in electrolyte-glucose-amino acid solution (pH 6.8, without digestive enzymes or bile) for 2 h, harvested by centrifugation, and transferred to fresh electrolyte-glucose-amino acid solution for an additional 3 h of incubation. The initial 2 h incubation served as the control for gastric juice exposure, while the subsequent 3 h incubation served as the control for bile juice treatment.

### Assessment of individual effects of pH and bile on probiotics' viability

The individual effects of pH and bile on probiotic tolerance were evaluated using LB, MRS, and YEPD media. To assess pH tolerance, the pH of the media was adjusted across a range from 1.5 to 9.5 in one-unit increments. A pH of 6.8 was used as the control condition. Bile tolerance was determined by supplementing the media with ox-bile at concentrations of 0.1%, 0.3%, 0.6%, 1%, and 2% (w/v), while unsupplemented media served as the control. 10⁸ cells/mL of *B. subtilis*, the *B. subtilis* mutants(*sinR* and *tasA eps*), and reference probiotic strains (*L. rhamnosus* GG and *S. boulardii*) were inoculated into pH-adjusted and/or bile-supplemented media and incubated at 37 °C for 4 h under mild agitation. Postincubation samples were serially diluted, plated on appropriate agar media, and viability was expressed as CFU/mL.^23^

### C. elegans survival assay

The wild-type *C. elegans* strain (N2) was used to evaluate the effect of probiotics on worm survival. Synchronized *C. elegans* eggs were prepared and transferred onto an NGM plate seeded with *E. coli* OP50 and incubated at 22 °C for 2 d. Subsequently, 25–30 L4-stage worms were transferred to NGM plates containing lawns of the test probiotic *B. subtilis*, its mutants *sinR* and *tasA eps*, or the reference yeast *S. boulardii*, each pre-cultured on NGM agar. Worm viability was monitored daily for up to three weeks using a dissection microscope, and live versus dead worms were recorded accordingly.^49^ Overall survival distributions were compared using the log-rank (Mantel-Cox) test and the log-rank test for trend, as implemented in GraphPad Prism. Median survival times were reported to indicate the time at which 50% mortality occurred.

### Colonization test in *C. elegans* gut

The L4 stage *C. elegans* worms cultured overnight on bacterial and/or yeast lawns were used in the experiment. A total of 12 ± 2 worms were randomly selected and washed 4–5 times with M9 buffer to minimize the presence of bacteria and/or yeast adhering to their cuticle. To further reduce surface-associated microbial cells, the worms were transferred to unseeded NGM plates and allowed to crawl for 5–10 min. Subsequently, worms were mechanically homogenized using a sterile pellet pestle, and the final volume was adjusted to 1 mL with M9 buffer. The resulting homogenate was serially diluted and plated on LB or YEPD agar to assess microbial load. CFU/mL were quantified. Additionally, images of microbial colonization were obtained using a Nikon TS-100 microscope equipped with Hoffman Modulation Contrast (HMC) optics and SPOT imaging software.^50^

### Assessment of intestinal integrity in *C. elegans* using the smurf assay

*C. elegans* worms were exposed overnight to test bacteria, *B. subtilis*, laboratory standard food of worms, *E. coli* OP50, a reference probiotic strain *S. boulardii*, and the positive control *C. albicans*, and washed three times with PBS to remove loosely associated microbes. The worms were then transferred to LB broth supplemented with 5% erioglaucine disodium salt (blue food dye) and *E. coli* OP50 at an optical density (OD₆₀₀) of 0.3 and incubated for 2–3 h to allow staining of the dye within the intestinal lumen. Following incubation, worms were immobilized using 2.5 mM levamisole and mounted on 1.4% agar pads. The intestinal barrier integrity was evaluated by counting worms that exhibited dye leakage into the body cavity (classified as Smurf phenotype) and those that retained the dye within the intestinal lumen (non-Smurf). Representative images were captured using Hoffman Modulation Contrast (HMC) optics on a Nikon TS-100 microscope equipped with SPOT imaging software. The percentage of worms displaying intestinal barrier disruption was calculated.^51^

### Congo red staining

LB agar was prepared without sodium chloride and supplemented with 50 µg/ml Congo red and 1 µg/ml brilliant blue- G20 dye. Appropriate dilutions of wild-type *B. subtilis* and mutants *sinR* and *tasA eps* were inoculated onto the plates. Plates were incubated at 37 °C for 48 h to allow colony development. Morphological differences and dye binding were documented using a Nikon SMZ1500 stereo microscope equipped with SPOT imaging software.^52^

### Biofilm production and quantification

Biofilm formation by *B. subtilis* and its mutant strains was assessed using LBGM medium. Bacterial cultures were adjusted to an OD_600_ of 0.3, and 10 µL of the bacterial suspension was inoculated into 1 mL of LBGM medium in 24-well plates. Following a 24 h incubation under static conditions, biofilms were carefully collected and transferred into microcentrifuge tubes. The biofilms were washed 3‒4 times with PBS, and the wet pellet was subsequently dried at 40 °C for 2 d. Biofilm biomass was quantified by measuring the dry weight, calculated as the difference between the initial and final weights of the tubes.

### Flocculation test

A cell suspension (OD_600_ –1.0) of wild-type *B. subtilis* and mutant strains (*sinR* and *tasA eps*) was prepared in sterile PBS (pH 7.4) and incubated at room temperature under static conditions for 3 h. Images were captured at 0 and 3 h to assess changes over time.^33^

### Agar adhesion and invasion assay

To evaluate agar surface adhesion, loopfuls of wild-type *B. subtilis* and the mutant strains (*sinR*, and *tasA eps*) were streaked onto LB agar plates and incubated at 37 °C for 24 h. Following incubation, the plates were exposed to a stream of tap water for 1 min. Colonies that remained intact after washing were considered adherent. Images of the colonies were captured both before and after washing to assess adhesion.

To examine the invasiveness of *B. subtilis* strains, all surface-adherent cells were gently removed using an L-shaped spreader under running water. The agar surface was subsequently inspected using a dissection microscope to visualize bacterial cells or biomass that had penetrated into the agar medium.^53^

### Soy milk preparation

A total of 500 g of soybeans were soaked in water overnight at room temperature. The soaked beans were then ground with 500 mL of Milli-Q water to obtain a slurry. Then, the mixture was filtered twice through muslin cloth to remove solids, yielding raw soy milk. The filtered soy milk was subsequently sterilized by autoclaving at 121 °C for 20 min. The soy milk was cooled and stored at 4 °C until further use in experimental procedures.

### Encapsulation of probiotic bacteria

Biofilm from wild-type *B. subtilis* was produced by supplementing LBGM medium with 50% (v/v) sterile soy milk, followed by static incubation at 37 °C for 24 h. The resulting biofilm was carefully harvested and washed 4‒5 times with Mili-Q water. To inactivate *B. subtilis* spores, the biofilm was autoclaved at 121 °C for 20 minutes and subsequently washed three additional times with sterile Milli-Q water. The wet weight of the biofilm was recorded and used for the subsequent procedure.

For encapsulation of probiotic cells, 10% (w/v) maltodextrin was used as the carrier matrix. This was mixed with either 10% or 20% (w/v) heat-treated biofilm or was left as maltodextrin alone as a control. The pH of all mixtures was adjusted to 6.8 ± 0.2. Subsequently, probiotic strains- *L. rhamnosus* GG, *Streptococcus thermophilus*, and *Escherichia coli* Nissle 1917- were inoculated into the sterilized matrix formulations at a final concentration of 10⁸ CFU/mL. A 10% (w/v) lactose formulation was used as a reference control in the study. Prior to lyophilization, samples were gradually cooled to −20 °C by placing them in a polystyrene insulated box. Lyophilization was conducted for 24–30 h using a laboratory freeze-dryer (Benchtop Freeze Dryer with Omnitronics, SP Scientific Inc.) at a vacuum pressure of 190 ± 15 mTorr with a condenser temperature set to approximately −100 °C.^5^ ^,^ ^54^

### Cytotoxicity assay

Caco-2 cells were cultured in Eagle's minimum essential medium (EMEM) supplemented with 10% fetal bovine serum (FBS) and incubated at 37 °C in a humidified atmosphere containing 5% CO₂. A suspension of Caco-2 cells (10^5^ cells/mL) was seeded into 24-well plates and incubated for two weeks to allow the formation of a confluent monolayer. After formation of the monolayer, 1, 10, and 50 mg of lyophilized biofilm powder were dissolved in the medium, then added to individual wells and incubated for 2 and 6 h at 37 °C in 5% CO2. After incubation, the cell monolayer was washed with PBS. An MTT assay was performed to assess cytotoxicity. Briefly, 300 µL of MTT working solution (0.5 mg/mL) was added to the biofilm-treated Caco-2 cell monolayer and incubated at 37 °C for 2 h. After incubation, the cell monolayer was washed again with PBS, DMSO was added to dissolve the formazan, and the samples were incubated for 5 min at room temperature. The dissolved formazan solution was subsequently measured at 570 nm using a microplate reader.

### Simulated gastric and bile tolerance test for encapsulated bacteria

To test the effect of gastrointestinal juices on biofilm-encapsulated probiotics, the encapsulated cells were inoculated into simulated gastric and bile juices (with the initial cell concentration maintained at 10^8^ cells/ mL) and incubated for 4 h at 37 °C. Viability was tested by the serial dilution method. A total of 10% maltodextrin-encapsulated and 10% lactose-encapsulated probiotics were used as the vehicle control and reference control, respectively.

### Gastric juice neutralization assay

To evaluate the gastric juice neutralization capacity, 100 ± 15 mg of lyophilized powders containing 10% or 20% heat-treated biofilm supplemented with 10% (w/v) maltodextrin, 10% or 20% heat-treated biofilm alone, and 10% maltodextrin (control) were individually exposed to varying volumes (2, 3, 5, and 10 mL) of simulated gastric juice at pH 2.50 ± 0.1. Finally, the pH of each mixture was measured using a calibrated pH meter (Accumet Basic, Fisher Scientific) to assess the buffering capacity of each formulation.

### Statistical analysis

Statistical analyses were performed using GraphPad Prism (GraphPad Software, San Diego, CA, USA). Data are presented as the mean ± standard deviation (SD). Comparisons among experimental groups were conducted using one-way analysis of variance (ANOVA) or two-way ANOVA and a *p*-value of  < 0.05 was considered statistically significant. Survival outcomes in *C. elegans* were analyzed using Kaplan-Meier survival curves, and differences between groups were assessed using the log-rank (Mantel-Cox) test, the log-rank test for trend, or the Gehan-Breslow-Wilcoxon test.

## Supplementary Material

Supplementary Material Final Supplementary file Gut microbes 1.docx

## Data Availability

All data supporting the findings of this study are included within the article and its supplementary materials. The raw data generated and analyzed during the current study are available from the authors upon reasonable request. No publicly available datasets were used in this study.
