# Supplementary material for: Biofilm overproduction enhances gastrointestinal stress tolerance and intestinal fitness in Bacillus subtilis
Source: Gut Microbes. 2026 Jun 21;18(1):2684066. doi: 10.1080/19490976.2026.2684066 (PMC13285563; doi:10.1080/19490976.2026.2684066)
Supplement: Supplementary Material — Final Supplementary file Gut microbes 1.docx [file KGMI_A_2684066_SM1428.docx]

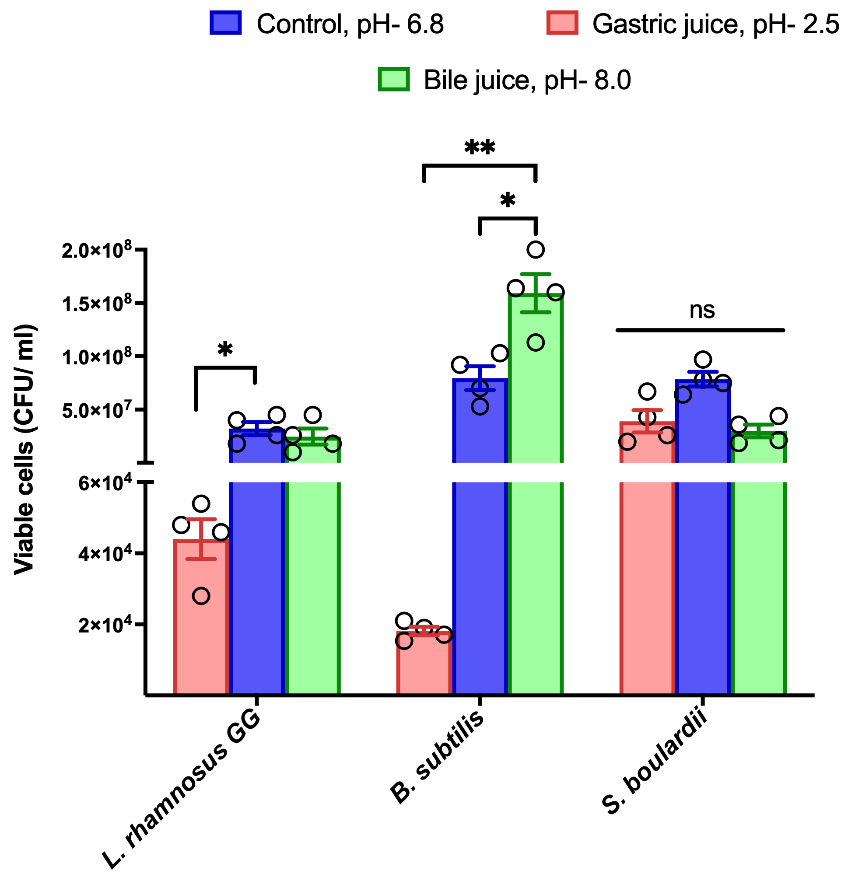


**Supplementary figure 1.** Gastric and bile juice exposed test probiotic, *B. subtilis* and reference probiotics, *L. rhamnosus* and *S. boulardii* for 24h of incubation.

**Supplementary table 1:** Individual effect of alkaline and acidic pH on viability of probiotics, *B. subtilis*, *L. rhamnosus* GG and *S. boulardii* for 4h of incubation; results are represented in CFU/ ml.

| STRAINS | ACIDIC pH | | | | CONTROL | ALKALINE pH | |
| --- | --- | --- | --- | --- | --- | --- | --- |
|  | 1.5 | 2.5 | 3.5 | 4.5 | 6.8 | 8.5 | 9.5 |
|  | Viability is expressed in x10⁷/ ml | | | | | | |
| *L. rhamnosus* | 0**** | 0.13 ± 0.0097**** | 14.47±  2.01**** | 22.22±  3.91** | 33.15±  4.28 | 24.12±  6.11 ns | 26.85±  4.78 ns |
| *B. subtilis* | 0.00020± 0.000083**** | 0.0067±0.0018**** | 0.011±  0.0033**** | 1.58±  0.15**** | 26.20± 3.97 | 18.80±  6.56* | 13.32±  2.52** |
| *S. boulardii* | 0.35±  0.15**** | 5.60±  1.58*** | 9.70±  1.51 (ns) | 9.00±  1.40 (ns) | 10.67±  1.18 (ns) | 11.87±  1.08 (ns) | 10.15±  2.09 (ns) |

**Supplementary table 2:** Effect of ox-bile on probiotic strains

| STRAINS | BILE CONCENTRATION | | | | | |
| --- | --- | --- | --- | --- | --- | --- |
|  | Control | 0.1% | 0.3% | 0.6% | 1.0% | 2.0% |
|  | Viability is expressed in x10⁷/ ml | | | | | |
| *L. rhamnosus* | 40.5± 11.47 | 21± 12.56 (ns) | 19.5± 9.03** | 10± 1.55** | 7.82± 2.19** | 3.71± 0.37*** |
| *B. subtilis*, WT | 43.5 ± 15.19 | 14.25± 6.80*** | 0.026± 0.020**** | 0.013± 0.012**** | 0.010± 0.0060**** | 0.00094± 0.00011 **** |
| *S. boulardii* | 14.65± 5.14 (ns) | 14.52 ± 5.28 (ns) | 11.65± 1.44 (ns) | 9.92± 2.51 (ns) | 11.97± 1.99 (ns) | 6.60± 3.37 (ns) |

**Supplementary table 3.** List of *B. subtilis* mutant strains used in the study.

| **Parent strain** | **Mutants** | **References** |
| --- | --- | --- |
| *B. subtilis*, parental strain NCIB 3610 | *tasA* | *Romero et al., 2010* |
| *B. subtilis*, parental strain NCIB 3610 | *kinA* | *McLoon et al., 2011* |
| *B. subtilis*, parental strain NCIB 3610 | *kinB* | *McLoon et al., 2011* |
| *B. subtilis*, parental strain NCIB 3610 | *kinC* | *McLoon et al., 2011* |
| *B. subtilis*, parental strain NCIB 3610 | *kinD* | *McLoon et al., 2011* |
| *B. subtilis*, parental strain NCIB 3610 | *slrR* | *Chai et al., 2009* |
| *B. subtilis*, parental strain NCIB 3610 | *eps* | *Romero et al., 2010* |
| *B. subtilis*, parental strain NCIB 3610 | *sinR* | *Kearns et al., 2004* |
| *B. subtilis*, parental strain NCIB 3610 | *tasA eps* | *Branda et al., 2006* |


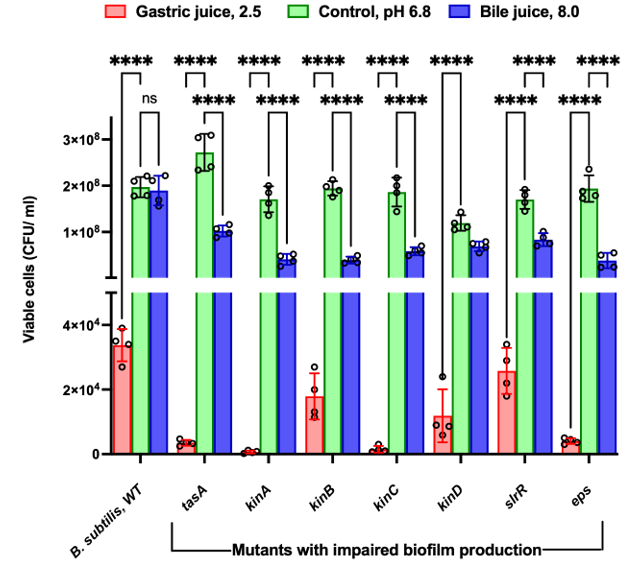


**Supplementary figure 2:** Wild strain, *B. subtilis*, and biofilm impaired mutant strains were exposed to gastric and bile juice for 4h incubation. The viability of these gastrointestinal juice exposed strains is represented in CFU/ ml. No statistically significant difference was observed between the wild-type and mutant strains following exposure to gastric juice.


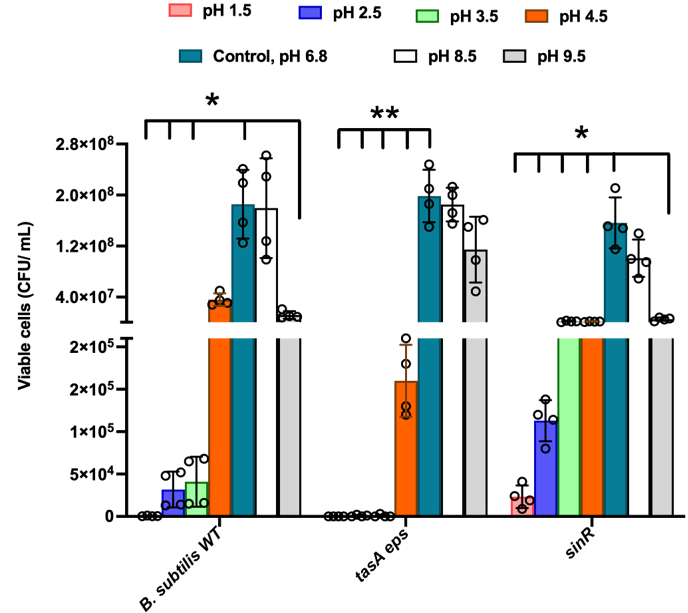


**Supplementary figure 3:** Effect of acidic and alkaline pH on wild strain, *B. subtilis* and mutants *sinR* and *tasA eps.* All the results are expressed in CFU/ml.


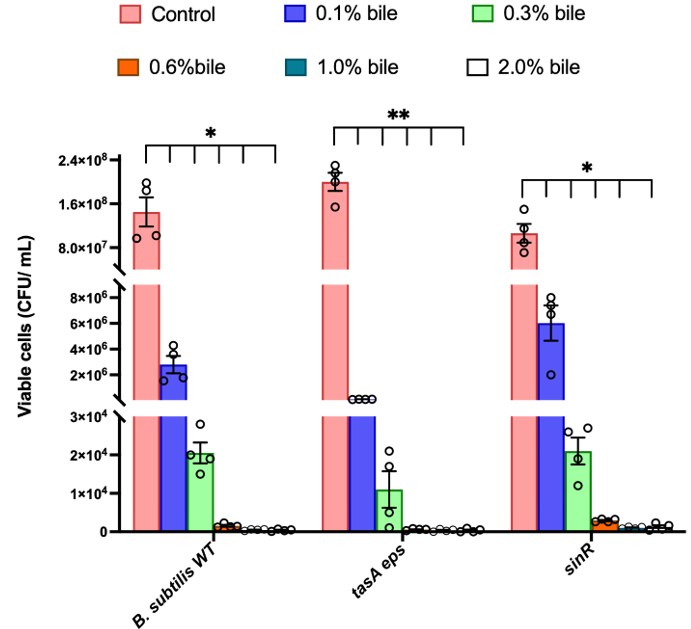


**Supplementary figure 4:** 0.1, 0.3, 0.6, 1 and 2% of bile supplemented to the growth media of wild strain, *B. subtilis* and mutants *sinR* and *tasA eps.* After 4h of incubation at 37°C, viability was checked. All the results are expressed in CFU/ml. Across all bile concentrations examined, no statistically significant differences were detected between the wild-type and mutant strains, *tasA eps* or *sinR*.


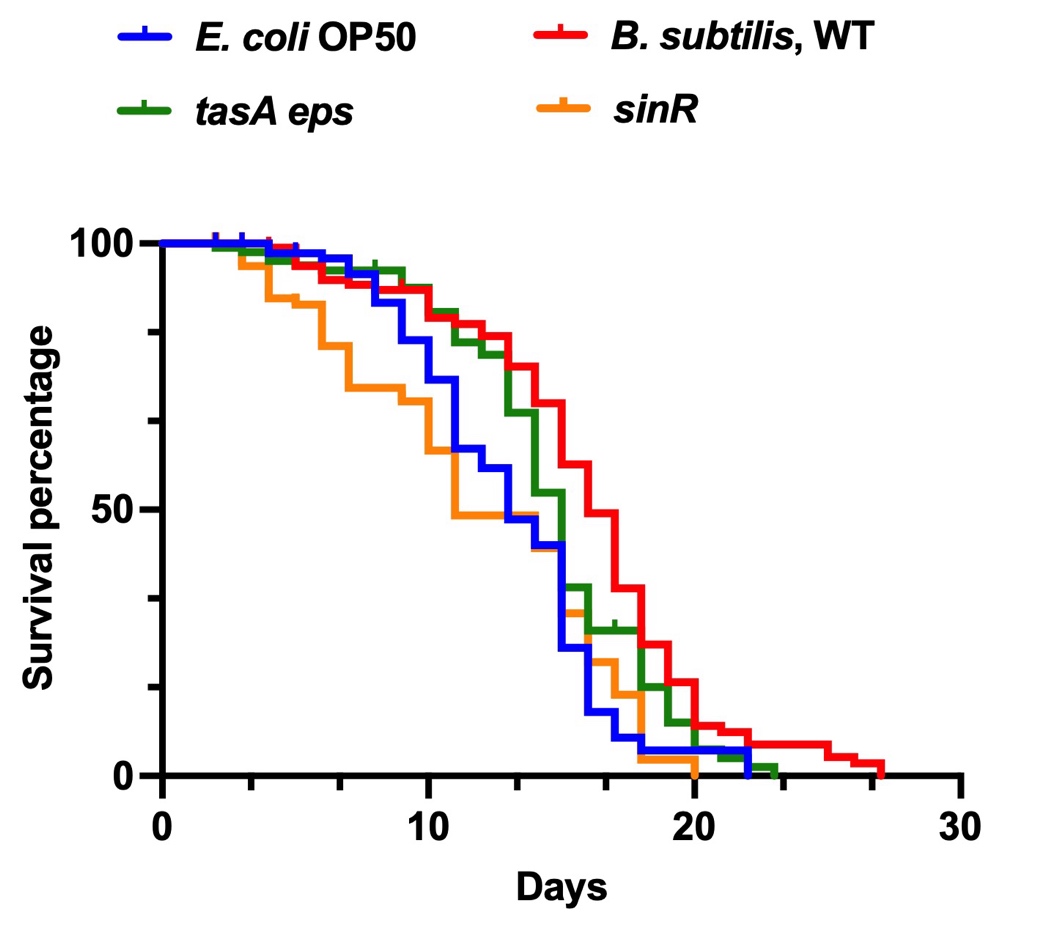


**Supplementary figure 5:** Survival percentage of worms that grown on wild strain, *B. subtilis* and mutants *sinR* and *tasA eps* lawn.


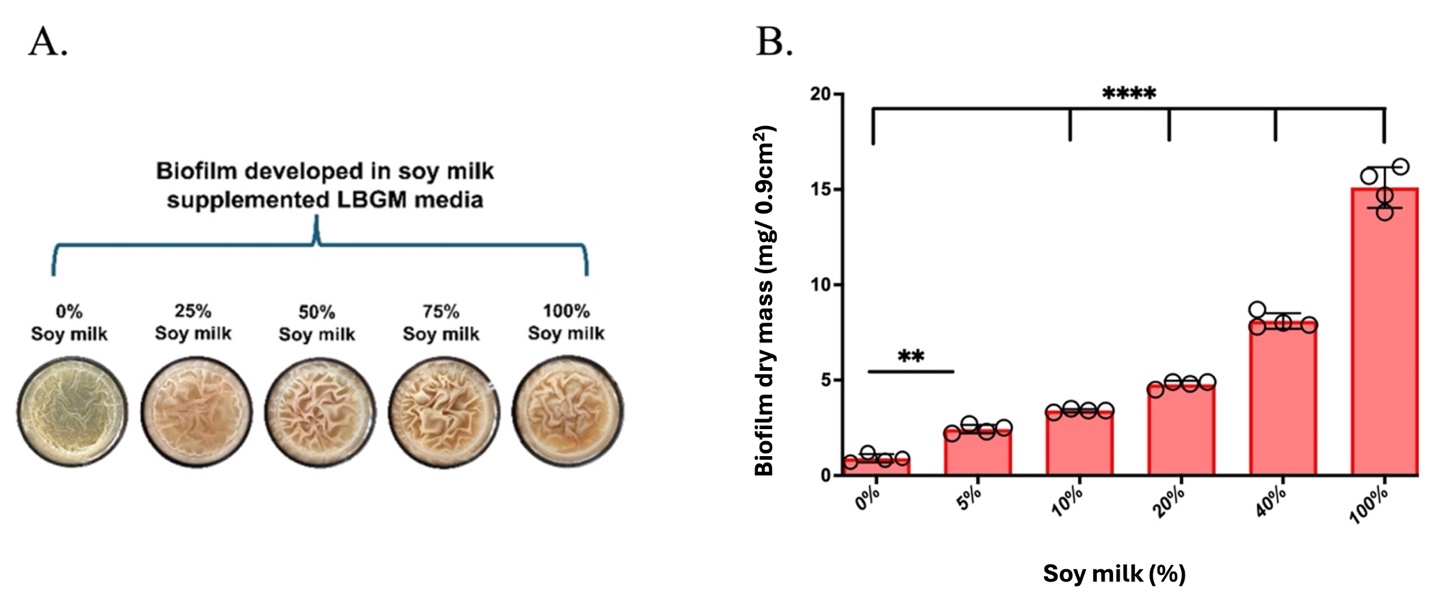


**Supplementary figure 6:** Effect of soymilk supplementation on *B. subtilis* biofilm formation. *B. subtilis* was cultured in LBGM media supplemented with 0, 25, 50, 75 and 100% soymilk. Biofilm formation increased with higher soymilk concentrations, as shown by thicker and denser biofilm layers at the air-liquid interface in the wells.


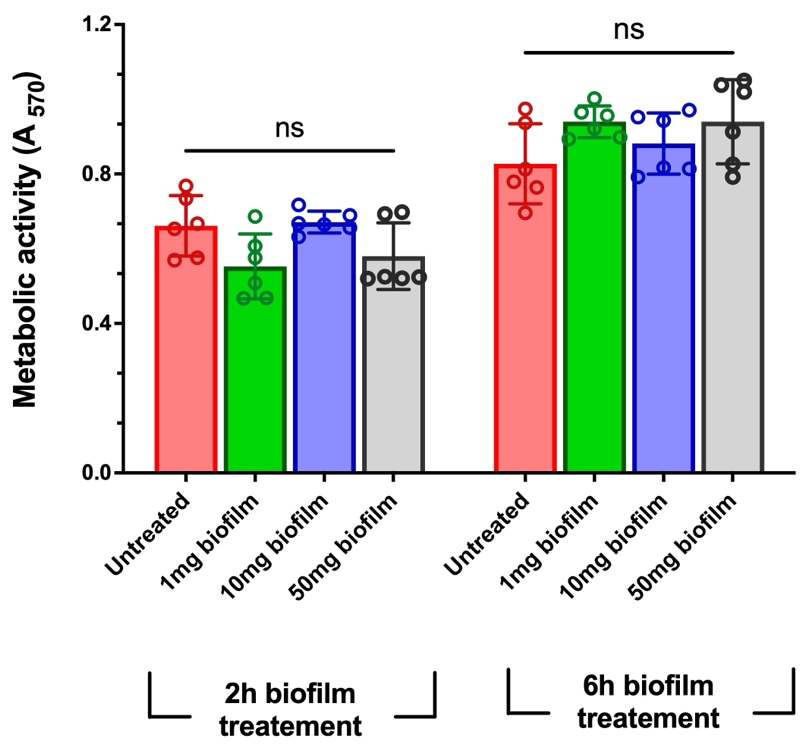


**Supplementary figure 7: Effect of *B. subtilis* biofilm on Caco-2 cell metabolic activity. Caco-2 cells were exposed to heat-killed biofilm at concentrations of 1, 10, and 50 mg for 2 and 6h intervals. Cell metabolic activity was assessed using the MTT assay, with absorbance measured at 570 nm.**
